# A Patient-Centered Mobile Phone App (iHeartU) With a Virtual Human Assistant for Self-Management of Heart Failure: Protocol for a Usability Assessment Study

**DOI:** 10.2196/13502

**Published:** 2019-05-23

**Authors:** Lingling Zhang, Sabarish V Babu, Meenu Jindal, Joel E Williams, Ronald W Gimbel

**Affiliations:** 1 College of Nursing and Health Sciences University of Massachusetts Boston Boston, MA United States; 2 School of Computing Clemson University Clemson, SC United States; 3 Department of Medicine Greenville Health System Greenville, SC United States; 4 Department of Public Health Sciences Clemson University Clemson, SC United States

**Keywords:** heart failure, mobile health, self-management, patient engagement, virtual human

## Abstract

**Background:**

Heart failure (HF) causes significant economic and humanistic burden for patients and their families, especially those with a low income, partly due to high hospital readmission rates. Optimal self-care is considered an important nonpharmacological aspect of HF management that can improve health outcomes. Emerging evidence suggests that self-management assisted by smartphone apps may reduce rehospitalization rates and improve the quality of life of patients. We developed a virtual human–assisted, patient-centered mobile health app (iHeartU) for patients with HF to enhance their engagement in self-management and improve their communication with health care providers and family caregivers. iHeartU may help patients with HF in self-management to reduce the technical knowledge and usability barrier while maintaining a low cost and natural, effective social interaction with the user.

**Objective:**

With a standardized systematic usability assessment, this study had two objectives: (1) to determine the obstacles to effective and efficient use of iHeartU in patients with HF and (2) to evaluate of HF patients’ adoption, satisfaction, and engagement with regard to the of iHeartU app.

**Methods:**

The basic methodology to develop iHeartU systems consists of a user-centric design, development, and mixed methods formative evaluation. The iterative design and evaluation are based on the guidelines of the American College of Cardiology Foundation and American Heart Association for the management of heart failure and the validated “Information, Motivation, and Behavioral skills” behavior change model. Our hypothesis is that this method of a user-centric design will generate a more usable, useful, and easy-to-use mobile health system for patients, caregivers, and practitioners.

**Results:**

The prototype of iHeartU has been developed. It is currently undergoing usability testing. As of September 2018, the first round of usability testing data have been collected. The final data collection and analysis are expected to be completed by the end of 2019.

**Conclusions:**

The main contribution of this project is the development of a patient-centered self-management system, which may support HF patients’ self-care at home and aid in the communication between patients and their health care providers in a more effective and efficient way. Widely available mobile phones serve as care coordination and “no-cost” continuum of care. For low-income patients with HF, a mobile self-management tool will expand their accessibility to care and reduce the cost incurred due to emergency visits or readmissions. The user-centered design will improve the level of engagement of patients and ultimately lead to better health outcomes. Developing and testing a novel mobile system for patients with HF that incorporates chronic disease management is critical for advancing research and clinical practice of care for them. This research fills in the gap in user-centric design and lays the groundwork for a large-scale population study in the next phase.

**International Registered Report Identifier (IRRID):**

DERR1-10.2196/13502

## Introduction

### Background

Heart failure (HF) is a chronic condition associated with significant morbidity, early mortality, and impaired quality of life (QoL), which poses tremendous economic and humanistic burden on patients, families, the health care system, and society [1]. Hospital readmission rates for HF are among the highest of any chronic disease [1,2]. Half of the patients with HF experience rehospitalization within 6 months of hospital discharge [3]. Those previously hospitalized for HF had the greatest rates of HF rehospitalization or cardiovascular death [4,5]. Limited access to care may be an additional problem among patients with limited socioeconomic means. Patients lacking access to primary care physicians after hospital discharge often experience clinical deterioration, necessitating hospital readmission [6]. This problem is especially magnified with uninsured patients, who may be forced into a cycle of relying on using costly emergency room visits [7].

According to the American College of Cardiology Foundation and American Heart Association guidelines for the management of HF, lack of improvement in health-related quality of life (HRQoL) after discharge from the hospital is a powerful predictor of rehospitalization and mortality [8-10], and no pharmacological therapy is a consistent determinant of HRQoL [8]. Previous studies have shown that self-management, which is expected to be integral to both the maintenance of wellness and the management of illness [11], can improve patients’ HRQoL [12-14]. As most chronic conditions are related to lifestyle, self-management was designed to meet the needs of managing daily treatment and life activities to improve health and health behaviors [11].

### Mobile Health and Heart Failure

To foster the ability of patients to practice more effective self-management and offset the difficulties due to lack of access to care, telemonitoring systems may be useful tools in reducing rehospitalization and improving QoL for patients with HF, as suggested by emerging evidence [1]. However, special digital devices required for telemonitoring are costly, limiting its potential for wide use, especially among low-income patients.

Optimal self-care is considered an important nonpharmacological aspect of HF management that can improve health outcomes [15]. Mobile health (mHealth) technologies have emerged as a way to actively engage patients in self-management and health care decision-making processes [16]. The access to mobile phones makes it possible for mHealth apps to transform treatment adherence through improved self-management [17]. About 85% of adults aged 65 years or above in the United States owned a mobile phone, and this proportion is increasing in all age groups [18]. The use of mobile phones to manage daily self-management of chronic diseases has been reported even among older adults with no experience in technology [14]. However, the dedicated, persistent use of self-monitoring systems and user engagement rates are still low [19,20]. Standardized, systematic mHealth usability assessments need to be performed, but even these have been insufficient in the past [21]. The Technology Acceptance Model [22] hypothesized that perceived usefulness, perceived ease of use, and attitude toward the technology were three factors determining whether the user would ultimately use or reject the technology. Patient involvement in the development of new information and communication technology (ICT) is crucial, given that the typical patient with chronic disease is an older adult with difficulties in understanding and using standard ICT equipment [23]. In order to support acceptance and later adoption of the new system, the innovation process should include the perspective of patients and the needs of other stakeholders in the care of chronic diseases [23].

### Virtual Human and Patient Engagement

**Figure 1:**
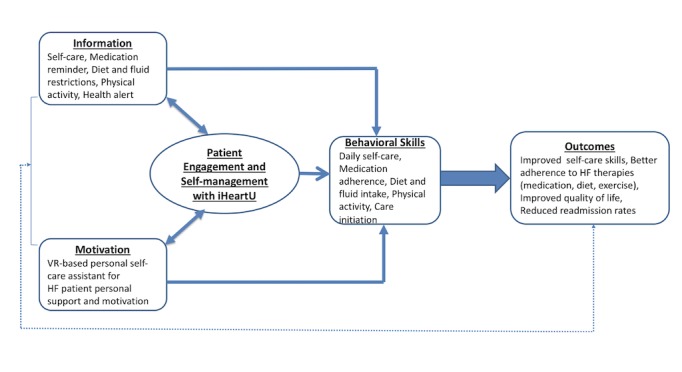
Conceptual framework for iHeartU: IMB model with patient engagement as mediator. HF: heart failure; VR: virtual reality.

To date, mobile phone–based apps for HF monitoring and management have not been widely researched. To address this gap, iHeartU—a mobile phone app with a novel virtual human assistant (“iHeartHelper”)—was developed to help patients with HF in their self-management by providing a holistic engagement experience (Figure 1). Its prototype has been undergoing usability testing since April 2018.

Patient engagement is a key mediator for behavior changes in patients with HF since patients who are engaged as decision-makers in their care tend to be healthier and have better outcomes (Figure 1) [17]. The development of iHeartU is based on the guidelines of the American College of Cardiology Foundation and American Heart Association for the management of HF and the validated Information, Motivation, and Behavioral skills behavior change model. This model emphasizes on information and knowledge about patient behavior, motivation to perform the behavior, and the patient’s behavioral skills [24].

A unique feature of this app—iHeartHelper—is an interactive virtual human or embodied conversational agent that resembles a human assistant and provides natural social interaction with users of the system. The personalized conversational interactions facilitated by the iHeartHelper can increase the engagement of patients with HF, health care providers, and family caregivers, which may lead to an improvement in patients’ health outcomes and reduction in hospital readmission rates.

There are existing studies that use virtual environment simulation to treat patients with posttraumatic stress disorder, fear of heights [25,26], and public speaking anxiety [27] and to conduct medical training [28]. However, whether the use of virtual human interface will improve patient engagement has not been systemically studied. The research assumption of this study is that a patient-centered mobile phone app with a virtual human assistant can improve engagement of patients with HF.

### Objectives

This study focuses on the user-centered design and assessment of the iHeartU mobile phone app. Unlike mHealth apps in previous studies, iHeartU is featured by the use of virtual human interface, which may involve more assessments in its usability testing but with the expectation of better user experience. Usability testing is the term used to describe the assessment of how easy a user interface is to operate [29]. The objectives of this study are two-fold: (1) to determine the obstacles to effective and efficient use of iHeartU among patients with HF and (2) to evaluate HF patients’ adoption, satisfaction, and engagement with regard to iHeartU.

## Methods

### Study Design

A single-arm prospective observation study is currently underway for the usability assessment of the iHeartU app. A mixed method design is used for this study, including several established usability evaluations through surveys, direct observations, interviews, think-aloud protocol, and focus groups. Additionally, the researchers have been working closely with the Patient Engagement Studio (an HF panel of patients and all stakeholders, including patients, family caregivers, physicians, nurses, and other health care providers) at Greenville Health System (GHS) to seek input and feedback on the design, development, and evaluation process.

### Participants

With the approval of the Institutional Review Board of GHS (Pro00066413), we aim to recruit 10 patients with HF to conduct the usability testing. The sample size is in accordance with the existing literature [30,31], where Kushniruk [30] and Vizri [31] have shown that 70% of severe usability problems can be discovered by the first five users and up to 85%, by the eighth user, following which less problems tend be identified, and these problems are also less significant. Patients are eligible to participate in this study if they have a clinical diagnosis of HF, are English-speaking, are able to operate study devices, have a New York Heart Association Functional Classification I-III, and are willing to provide informed consent for participating in the study. Patients are recruited by their direct care team during scheduled clinic visits. They are informed about the purpose of the study upon providing written consent for participation. There are no expected risks or discomforts in the study other than possible confusion when using iHeartU. Participation in this study is completely voluntary; therefore, patients can refuse to participate or withdraw from the study at any time. Patients who are unable to operate the devices and have a limited cognitive ability, as determined by the patient’s clinician, will be excluded from consideration.

Health care providers in the direct care team for patients with HF, including physicians and nurses, are also involved in the testing. One representative of each group is asked to complete the survey questionnaires after providing consent. The research team collects their feedback and input to provide a helpful clinical perspective for improving the design of the mobile phone app.

### Measures

An iterative approach is used to refine iHeartU for patients with HF based on their experience of interacting with this app while aiming for continual patient satisfaction. Patients will be the primary users of the app. Health care providers will help identify patient needs and health problems. The following instruments and methods will be used to collect data:

- A demographic and background questionnaire to describe personal and health information as well as experience with mobile phone use.
- Usability evaluation metrics, guided by the International Organization for Standardization 9241-11 Usability framework and mobile health usability research [21], to evaluate effectiveness, efficiency, and satisfaction. Engagement will be added as the fourth dimension (Table 1).

- Think-aloud protocol to capture patients’ cognitive processes while performing representative tasks on the app [30].
- Individual interviews with open-ended questions to evaluate task-specific user satisfaction regarding what study participants think about the interface and functions of the app as well as any specific issues that they find confusing.
- Networked minds survey to evaluate social presence in terms of communication [32] when the user interacts with the virtual human assistant.
- System usage logs to quantify patient engagement with the system and guide improved app design and development [33] and nonscheduled patient-initiated feedback to indicate patient satisfaction and engagement.
- Focus groups to define the essential expectations and needs that are personally and clinically relevant and to achieve a consensus on the key features and functions of iHeartU and the overall mobile self-management system.

### Procedure

The usability assessment will follow the phases below and last for about 17-24 months.

#### Enrollment Phase

A panel of patients, family caregivers, and health care providers was hosted in the GHS Patient Engagement Studio to provide initial thoughts and suggestions on the preliminary product of iHeartU. A meeting with a group of health care providers for patients with HF was also held during the prototype development for advice on the design. The eligible patients with HF were introduced for enrollment by their direct care team at GHS.

#### Intervention Phase

HF patient representatives and health care providers who care for patients with HF complete the demographic and background questionnaire, navigate the features of iHeartU, and test its functions along with the use of supplemental devices. Users perform predefined tasks by the research team and conduct interactive conversations with the iHeartHelper to input clinical variables: weight; systolic and diastolic blood pressure; heart rate; and subjective reports for medication adherence, dietary sodium and fluid intake, and physical activity. In case of any technical difficulty, patients can always choose to enter the data manually. This assessment is conducted by using usability evaluation metrics, the think-aloud protocol, individual interviews, and networked minds survey. The smartphone and supplemental devices are provided by the research team.

#### Follow-Up Phase

Iterative usability testing with the improved iHeartU app is conducted and evaluated among patients after their use at home. The usability regarding patient engagement is examined by using system usage logs and the User Engagement Scale. A focus group discussion is organized with the patient participants to further identify the essential needs and desirable features and functions of iHeartU to improve patient satisfaction and engagement.

### Data Analysis

Quantitative analysis will be performed on the survey data. The descriptive statistics will be summarized on patients’ background and experience. The significance of overall System Usability Scale [26] will be assessed by using the Student *t* test [37,38]. The User Engagement Scale [36] and Networked Minds survey [32] will be used for analysis. The significance of iHeartU use will also be assessed by comparing the ratio of observed use to expected use to the hypothetical mean of 1.0 by using the Student *t* test [38]. The interrater agreement of raw system usability scores will be assessed by the intraclass correlation coefficient with 95% CIs [39]. The Cronbach alpha test will be performed on the data to evaluate the internal consistency of the responses.

**Table 1:** Usability evaluation metrics.

Aim	Measure	Method
Effectiveness	Task completion: with ease, with minor mistakes, or failure Error coding	Direct observation
Efficiency	Time used to complete a task	Count
Satisfaction	System Usability Scale [34] Poststudy Usability [35]	Questionnaire
Engagement	User Engagement Scale [36] System usage log	Questionnaire Built-in tracker

Qualitative analysis will be performed on the data collected through think-aloud protocol, individual interviews, and focus groups by using the Grounded Theory to identify emerging themes directly from patients’ own words and thoughts. Each think-aloud protocol will be reviewed line-by-line by two researchers in order to reach a consensus on the coding. The ease of use will be assessed and coded by scrutinizing the recorded details (such as facial expression and finger movement) of patients’ performance on using iHeartU. Individual interviews and focus group discussions will be analyzed using the constant comparative method. The transcript will be reviewed and coded for recurrent themes independently by two researchers. Themes will be combined by agreement of the two researchers who will create theme tags based on the recurrent themes, and the transcripts will be rereviewed collaboratively by the two researchers in order to tag all instances of the themes. Major themes and subthemes will be developed via an iterative review process.

## Results

### Development of iHeartU

**Figure 2:**
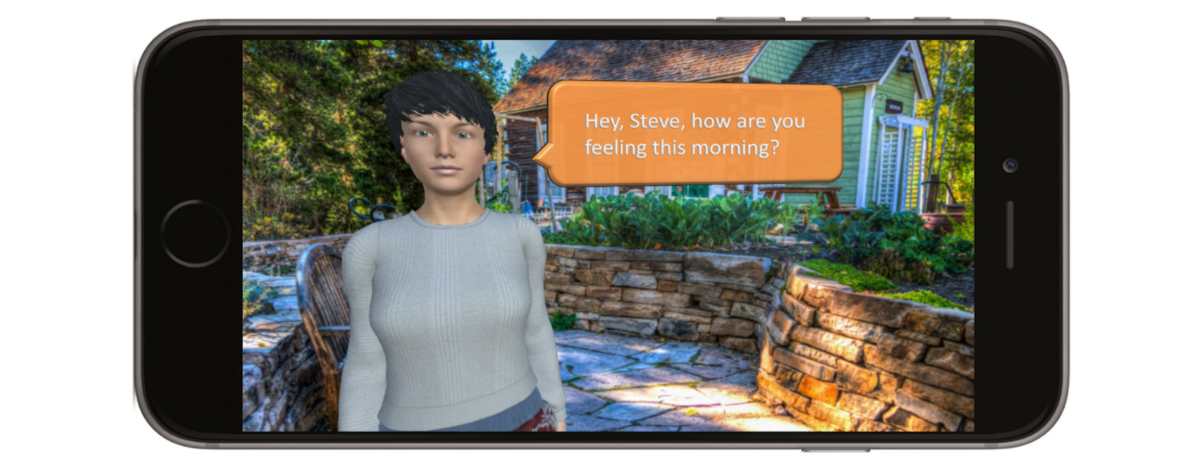
Example of the interface of iHeartHelper.

The development of iHeartU started in May 2017 with developmental approval from the Office of Compliance at Clemson University. Institutional review board approval was obtained (#Pro00066413) from the GHS in October 2017. The prototype of the mobile phone app has been produced. The screenshot of its interface with the iHeartHelper is indicated in Figure 2. A variety of outfits for the iHeartHelper and background options are available for random picking every time when the user opens the app. Key self-care behaviors, including adherence to prescribed HF therapies such as medications, dietary sodium and fluid restrictions, and exercise, are built into the daily checkup page [40]. Besides these HF therapies, patients are taught to recognize and manage changes in symptoms and seek health advice when such changes occur.

The iHeartHelper proactively engages the patient at times prescribed by the clinician; requests the patient to provide a report of their general progress, medications, activity, and other behavioral aspects via natural dialogue; and records the patient’s responses as audio files. Real-world interactions between physicians and patients were shadowed at the GHS Heart Failure Clinic by the research team and used to enrich the conversation scenarios with the iHeartHelper. The sample scripts are demonstrated in Textbox 1. A closed caption is installed to meet the needs of patients who may have hearing impairment. The iHeartHelper’s speech speed can be customized as per the patients’ preference.

**Figure 3:**
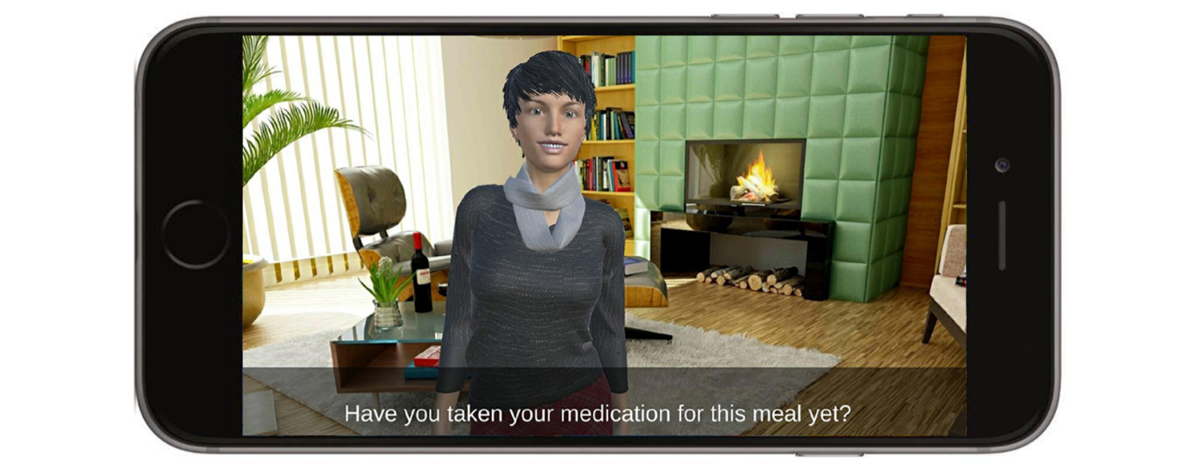
Example of daily check-up and monitoring by the iHeartHelper.

**Figure 4:**
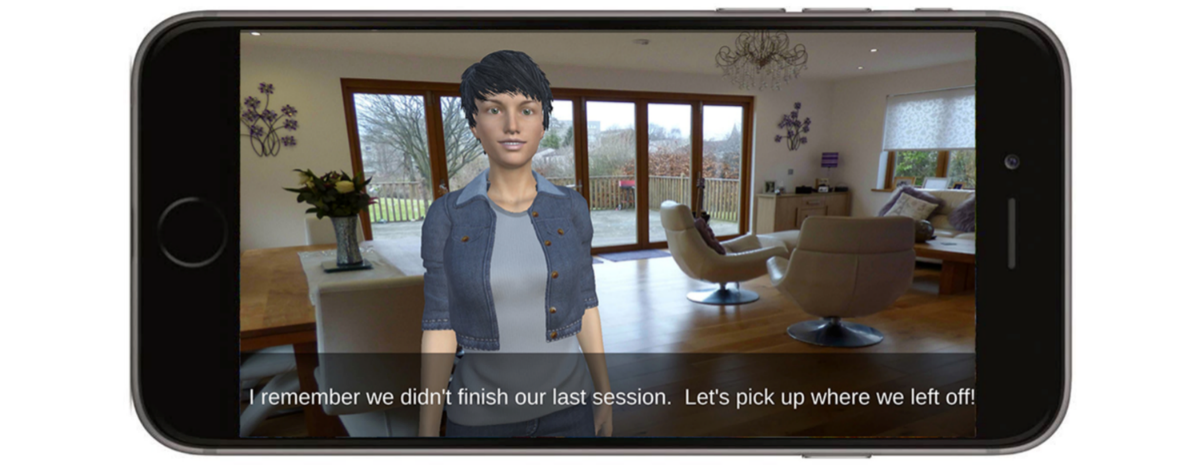
Example of follow-up and notifications by the iHeartHelper.

The iHeartHelper serves as a health assistant who checks on the health of the patient three times daily and records vital signs, diet, fluid intake, weight, and any symptom that the patient would like to report. The objective data are manually input by the patient in the current development phase, with error checking for a numeric format. Patients receive a push notification to remind them to open the app when it passes the preset time. Patients have the facility at any time to convey messages and alerts to their health care provider and caregivers by reporting issues to the iHeartHelper through audio recording. Two representative screenshots are illustrated in Figures 3 and 4.

**Figure 5:**
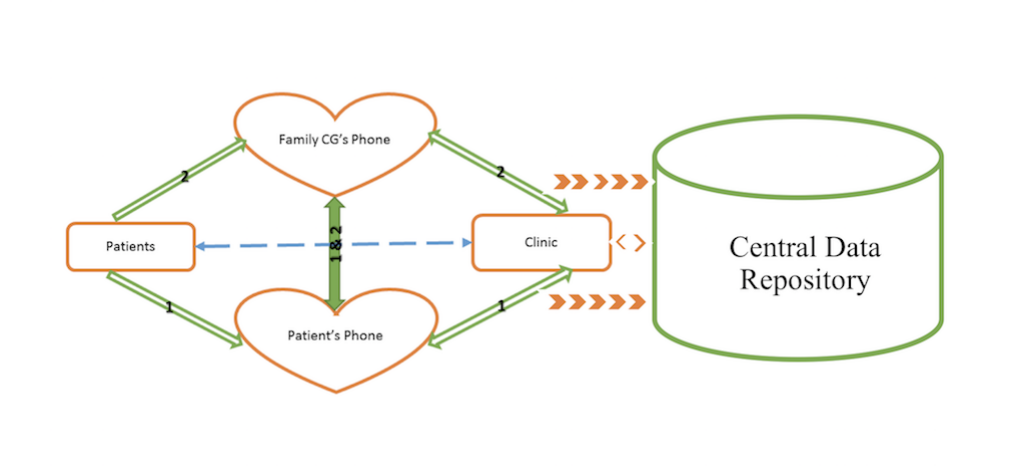
Data transfer structure for iHeartU patient-centered mobile self-management system. CG: caregiver.

**Figure 6:**
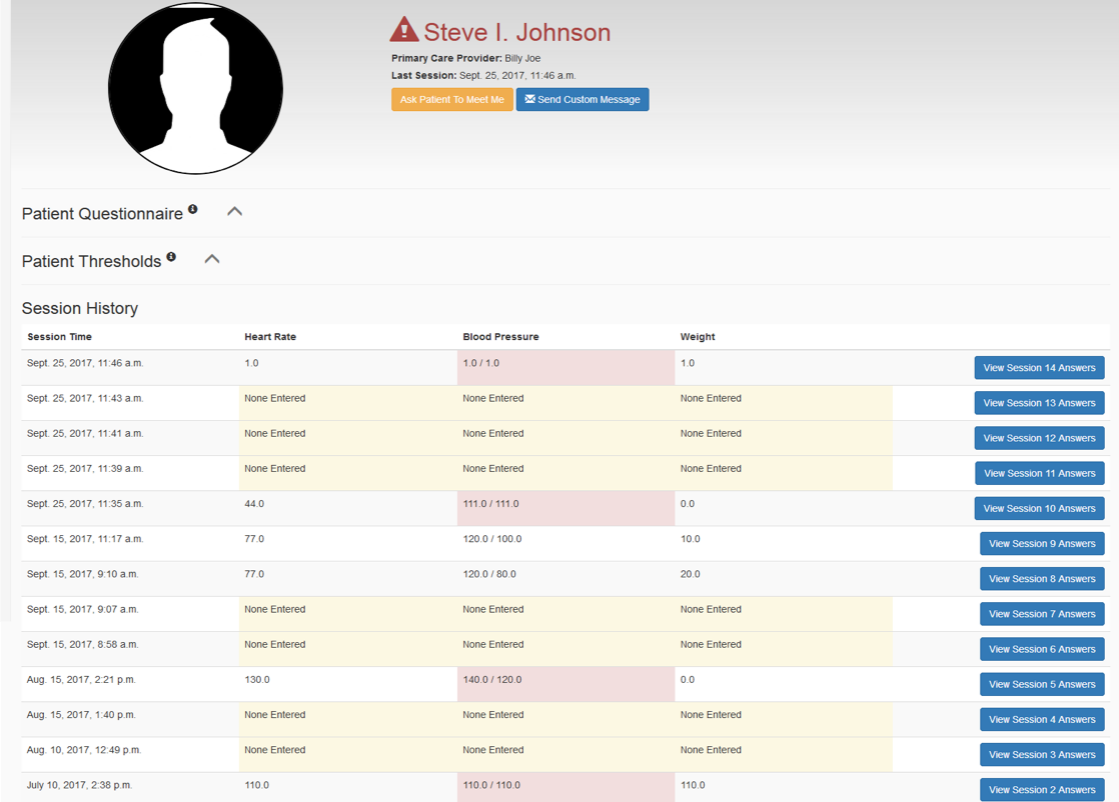
Screenshot of the Web portal for health care providers.

Audio files as well as other data can be periodically uploaded by a reporting module to a central data repository, which allows the health care provider to monitor the patient’s progress as needed via a Web-based interface (Figure 5). The Web portal for health care providers has also been developed and linked with the iHeartHelper (Figure 6). The audio files and any information (eg, vital signs and weight) recorded by the patient can be accessed by the care team (eg, physician, physician assistant, nurse, and disease manager). Once the patient’s data exceed the predefined thresholds with respect to the clinical guideline and physician’s suggestions, the health care provider will be alerted. According to the severity of the situation, the health care provider can send a message to the patient through the portal to give him/her suggestions or schedule a phone call or visit. The message is delivered to the patient by the iHeartHelper. There are two-stage confirmations to ensure that the message is delivered to the patient.

**Sample scripts of the conversation scenarios between the virtual human assistant (iHeartHelper) and the patient.**

Have you had anything to eat since we last talked?

- Yes - What did you have to eat?
- No - Please be sure to eat three times daily!

What fluids have you had to drink today and how much of each did you drink? Have you taken your medication for this meal yet?

- Yes - Great!
- No - Please remember to take your medications at every meal.

Have you done any exercise since our last session?

- Yes - what kinds of activities did you do?
- No - Alright, try to do a little bit of exercise every day.

Have you felt any shortness of breath since we last spoke?

- Yes - Can you please describe the shortness of breath and when it happened?
- No - Okay, good! Remember to always alert your doctor immediately if you feel any shortness of breath.

Do you have any other symptoms you would like your doctor to be aware of?

- Yes - What are the symptoms that are worrying you?
- No - Okay, please keep your doctor informed if you experience any unexpected symptoms.

### Usability Assessment

Two panel meetings with all key stakeholder representatives, including patients, family caregivers, and health care providers, have already been conducted to collect inputs and suggestions at the GHS Patient Engagement Studio before and during the development of iHeartU. The feedback was also solicited from a meeting with health care providers in the Department of Internal Medicine and the Department of Cardiology at GHS. The suggestions from the stakeholders and health care providers were incorporated into the iHeartU development for usability testing. Enrollment of study participants began in January 2018. As of September 2018, the preliminary data collection in the intervention phase has been completed. The follow-up phase is expected to be completed and the entire usability testing is expected to be concluded by the end of 2019. The data will be analyzed accordingly, and the results will be submitted for publication upon completion of the study. Two representatives of health care providers will be invited to conduct a usability assessment following the same process as patients and provide inputs from their perspectives. Their feedback will serve as important supplemental information to guide further improvement on iHeartU. Thus far, testing has been completed by a physician in the direct care team.

## Discussion

### Uniqueness of iHeartU

The main contribution of this work is the development of an HF patient–centered self-management system with a virtual human interface, which may support patients’ self-care at home and strengthen communications among patients, health care providers, and family caregivers for better care transition and coordination. To our knowledge, this is the first mobile phone app with a virtual human interface for HF self-management.

There are several foreseeable advantages to this design of iHeartU. First, the virtual human assistants can carry out conversations proactively to record a daily log for patients’ self-management behaviors. Since most patients with HF are older adults with relatively lower rates of technology adaption, the intuitive voice-based data collection and easy-to-use features are better than the features of traditional mobile apps that require patients to manually input data. The interactive process aims to resemble the clinical workflow in reality but be more proactive with closer monitoring on patients. Second, the virtual human interface may provide more psychological comfort and companion to patients with HF by sharing news, weather, and jokes with patients to make daily conversions more engaging and socially motivating. Third, the virtual human interface provides personalized health coaching through virtual “face-to-face” communications using education materials tailored for patients’ individual situation. Fourth, the virtual human interface serves as a digital personal assistant to remind patients of their daily medication use, which may improve patients’ compliance to HF therapies. This virtual assistant can also remind patients of their physician office visits and facilitate their follow-up visits. This feature will be expected to expand their accessibility to care, improve care coordination, and reduce the cost incurred due to unnecessary emergency visits or readmissions, especially for low-income patients with HF. Fifth, data collection via this mobile self-management system in community settings may lay the groundwork for future clinical studies and big data analysis.

### Further Development of iHeartU

Besides the embedded functions described above, the Bluetooth-enabled devices (eg, weight scale and blood pressure monitor) will be integrated into iHeartU. For patients with these Bluetooth sensors, data will be automatically transmitted to iHeartU. They can also choose to record their data with validation and error checking. The manual input option will be still available as a backup.

A personalized customization on the iHeartHelper such as gender, race, and age will be developed. The entertainment features will also be embedded in the app. The iHeartHelper can talk about jokes and report the weather and news as per the user’s commands. Such interaction may resemble the interaction of the user with Alexa and Siri. The iHeartHelper will put on an outfit according to the weather change as well, for instance, wearing a rain coat when it is raining.

During the usability assessment, besides testing the close-to-finish product, a list of desirable features will be obtained from the end users to know their thoughts and solicit feedbacks. Thereafter, further development and improvement will be undertaken and tailored to the needs of patients based on the outcome of the usability testing.

The Web portal for health care providers will be further enhanced. In addition, the mobile phone app for family caregivers will be developed. The caregiver app will show the real-time patient’s data (with the patient’s consent) and receive messages from the patient’s health care providers, so that he/she can help monitor the patient’s health. An overall assessment will be conducted on the entire mobile self-management system after its full development.

### Conclusions

Findings from the standardized usability testing on iHeartU will be used to refine its preliminary design and contents, which may enhance its implementation and dissemination in the later phases. Such a usability testing model can be translational and adapted to other health management programs through disease-specific customization with the involvement of patients and key stakeholders. Further research is needed to establish a standardized, systematic mHealth usability assessment for chronic HF patient care, which can improve methodological consistency and make it possible to compare findings across different mHealth app evaluations [41].

## Appendix

Multimedia Appendix 1 Peer-review report from Greenville Health System.

## Abbreviations

GHS: Greenville Health System
HF: heart failure
HRQoL: health-related quality of life
ICT: information and communication technology
mHealth: mobile health
QoL: quality of life
