# Supplementary material for: A Patient-Centered Mobile Phone App (iHeartU) With a Virtual Human Assistant for Self-Management of Heart Failure: Protocol for a Usability Assessment Study
Source: JMIR Res Protoc. 2019 May 23;8(5):e13502. doi: 10.2196/13502 (PMC6552454; doi:10.2196/13502)
Supplement: Multimedia Appendix 1 [file resprot_v8i5e13502_app1.pdf]

ZHANG FEEDBACK

1:

Reviewed but did not provide comments

2:

Strong proposal for a usability study of an mHealth heart failure tool, which would improve patient self-care and reduce ED utilization and readmissions--cited nationally by CMS as a key area for improvement across national health systems. Study methodology is well-outlined with mixed methods approach and clear timeline. Incorporates multiple perspectives for this study phase--provider, patient and scholarly baseline. One caution--the GHS ED utilization numbers for the uninsured population are not necessarily heart failure patients.

3:

Very well written and thought-out proposal.

4:

Excellent chance to external funding possibilities; provide some clarity for what the IHeart app does and interface between this and provider (not part of this proposal but would be key to future applicability)

PURPOSE

Strengths

- The overall idea of creating a user-friendly tool to improve self-management for heart failure patients is strong and has the potential to greatly improve population health. As stated below, the exact scope of the project is unclear and could benefit from a narrowed focus or clarification in how it is presented.
- If successful, could make important contribution given the burden of HF, both for patients and their families and for the health care system and public health.
- Clear goals and aims outlined, including part this seed study plays in larger study. Aim 2 is important in patient-centered research, but often overlooked. Clear, achievable deliverables described.

Weaknesses

- The goal of the project is not stated clearly - is it it develop iHeartU or to test its feasibility and effectiveness, or both? If the tool is not already developed, it seems like the first step should be to gather data to create the tool and then test it. Developing the tool in and of itself appears like a lofty goal.
- Assess and Evaluate used without any definition of terms used: e.g., effectiveness, effort and resource, satisfaction, etc., or specific measures or approaches to assess or evaluate.

Appears in Design & Methods, but a sentence here would be helpful. Where will low income HF patients get smartphones and service plans?
- First NIH proposal timing seems too close to seed grant start; unsure if any results from seed money could support proposal at that time. Second NIH proposal seems more realistic.

BACKGROUND AND SIGNIFICANCE

Strengths
- The authors have done a nice job of summarizing the problem at hand and the potential application of mobile tools to improve health outcomes.
- Clearly lays out extensive impact and burden of HF. Demonstrates attention to some potential barriers, and intends to use patient input in design and initial evaluation of device and program.
- Strong literature review clearly states the scope of the problem and locates the current study as a contribution to the field. HF is considered one of the conditions in which care guidelines are supported most strongly by evidence, thus lending itself to automation with less risk. Arguments for appropriateness of mobile technologies and mhealth challenges are convincing and correspond well to my knowledge of the literature.

Weaknesses
- As stated above with regards to the purpose statement, the scope of the project appears to large and/or diffuse. A narrowed focus or clarity in writing is needed.
- ED visits per 1,000 uninsured adults was 824.4% doesn't make sense. Was it 1K uninsured HF patients? How can it be 824.4%? How will low income people manage cost of phones capable of program as well as monthly service? Exclusion of those with limited cognitive ability may limit population who could benefit most.
- "Virtual human" is not defined clearly when needed (i.e., until WAY down at the end--finally found it!), but is a vital concept in the proposed study. "Conversations" and inserting jokes or weekly news are specifics, not general concepts related to virtual human. However, embodied conversational agent meant to provide natural social interaction is MUCH better.

RESEARCH DESIGN AND METHODS

Strengths
- The authors have chosen a mixed-methods approach using a variety of standard instruments.
- Use of patient input in ongoing assessment. Familiarity with usability evaluation and mixed-method approaches. Clear description of complex process, instruments, types of data collected, and types of analysis appropriate to the data type and sources. Solid grounding in theory of behavior change. Excellent use of PES. Iterative process allows for ongoing feedback.
- Budget justification is strong (given word limits and compared to other proposals I reviewed). Sample size, inclusion/exclusion criteria are well defined. Inclusion of clinicians is important and appropriate; would like to see some physicians AND nurses

(perhaps home health nurses, who spend a lot of their time educating and motivating patients, in the provider mix). First proposal with a timeline! Terrific.

Weaknesses

- The translation of the findings of the data analysis to how they will specifically refine the proposed tool need clarified. There is a bit of a disconnect throughout the entire proposal that could benefit from a narrowed focus and clearly written and consistent goals and hypotheses.
- None noted beyond those already mentioned concerning cost to low-income patients and exclusion of persons with limited experience with smart phone technology, even if a caregiver is present to assist.
- Study design is more a list of concepts and approaches, not an actual design. Unclear how 30 recruited patients will be selected for representativeness. Unclear how qualitative analysis (2nd paragraph in data analysis section) would contribute to study. The process of analysis was clear, but what do the researchers and app developers DO with the verified and prioritized terms and themes developed? How will that process contribute to the app development or revisions is not clear.
